# Hemoglobin A_1c_ variability as an independent correlate of cardiovascular disease in patients with type 2 diabetes: a cross-sectional analysis of the Renal Insufficiency and Cardiovascular Events (RIACE) Italian Multicenter Study

**DOI:** 10.1186/1475-2840-12-98

**Published:** 2013-07-05

**Authors:** Giuseppe Penno, Anna Solini, Giacomo Zoppini, Emanuela Orsi, Cecilia Fondelli, Gianpaolo Zerbini, Susanna Morano, Franco Cavalot, Olga Lamacchia, Roberto Trevisan, Monica Vedovato, Giuseppe Pugliese

**Affiliations:** 1 Department of Endocrinology and Metabolism, University of Pisa, Pisa, Italy; 2 Department of Internal Medicine, University of Pisa, Pisa, Italy; 3 Division of Endocrinology and Metabolic Diseases, University of Verona, Verona, Italy; 4 Endocrinology and Diabetes Unit, Fondazione IRCCS “Cà Granda - Ospedale Maggiore Policlinico”, Milan, Italy; 5 Diabetes Unit, University of Siena, Siena, Italy; 6 Complications of Diabetes Unit, Division of Metabolic and Cardiovascular Sciences, San Raffaele Scientific Institute, Milan, Italy; 7 Department of Internal Medicine and Medical Specialties, “La Sapienza” University, Rome, Italy; 8 Unit of Internal Medicine, Department of Clinical and Biological Sciences, University of Turin, Orbassano, Turin, Italy; 9 Unit of Endocrinology and Metabolic Diseases, University of Foggia, Foggia, Italy; 10 Diabetes Unit, Hospital of Bergamo, Bergamo, Italy; 11 Department of Clinical and Experimental Medicine, University of Padua, Padua, Italy; 12 Department of Clinical and Molecular Medicine, “La Sapienza” University, Rome, Italy

**Keywords:** Chronic kidney disease, Retinopathy, Hemoglobin A_1c_, Risk factors, Type 2 diabetes

## Abstract

**Background:**

Previous reports have clearly indicated a significant relationship between hemoglobin (Hb) A_1c_ change from one visit to the next and microvascular complications, especially nephropathy (albuminuria and albuminuric chronic kidney disease, CKD). In contrast, data on macrovascular disease are less clear. This study was aimed at examining the association of HbA_1c_ variability with cardiovascular disease (CVD) in the large cohort of subjects with type 2 diabetes from the Renal Insufficiency and Cardiovascular Events (RIACE) Italian Multicenter Study.

**Methods:**

Serial (3–5) HbA_1c_ values obtained during the 2-year period preceding recruitment, including that obtained at the enrolment, were available from 8,290 subjects from 9 centers (out of 15,773 patients from 19 centers). Average HbA_1c_ and HbA_1c_ variability were calculated as the intra-individual mean (HbA_1c_-MEAN) and standard deviation (HbA_1c_-SD), respectively, of 4.52±0.76 values. Prevalent CVD, total and by vascular bed, was assessed from medical history by recording previous documented major acute events. Diabetic retinopathy (DR) was assessed by dilated fundoscopy. CKD was defined based on albuminuria, as measured by immunonephelometry or immunoturbidimetry, and estimated glomerular filtration rate, as calculated from serum creatinine.

**Results:**

HbA_1c_-MEAN, but not HbA_1c_-SD, was significantly higher (*P*<0.0001) in subjects with history of any CVD (n. 2,133, 25.7%) than in those without CVD (n. 6,157, 74.3%). Median and interquartile range were 7.78 (7.04-8.56) and 7.49 (6.81-8.31), respectively, for HbA_1c_-MEAN, and 0.47 (0.29-0.75) and 0.46 (0.28-0.73), respectively, for HbA_1c_-SD. Logistic regression analyses showed that HbA_1c_-MEAN, but not HbA_1c_-SD (and independent of it), was a significant correlate of any CVD. Similar findings were observed in subjects with versus those without any coronary or cerebrovascular event or myocardial infarction. Conversely, none of these measures were associated with stroke, whereas both correlated with any lower limb vascular event and HbA_1c_-SD alone with ulceration/gangrene. All these associations were independent of known CVD risk factors and microvascular complications (DR and CKD).

**Conclusions:**

In patients with type 2 diabetes, HbA_1c_ variability has not a major impact on macrovascular complications, at variance with average HbA_1c_, an opposite finding as compared with microvascular disease, and particularly nephropathy.

**Trial registration:**

ClinicalTrials.Gov NCT00715481

## Background

Glycemic exposure, i.e. the extent and duration of hyperglycemia, is a major risk factor for both micro and macrovascular complications of diabetes. In fact, data from subjects with type 1 and type 2 diabetes from the Diabetes Control and Complications Trial (DCCT) and the UK Prospective Diabetes Study, respectively, have shown a strong association between exposure to hyperglycemia and diabetic complications [1,2] as well as a significant benefit of intensive glycemic control on the development and progression of microvascular disease [3,4]. In addition, post-trial, prolonged follow-up has uncovered a beneficial effect of prior intensive regimen on cardiovascular disease (CVD) in both studies [5,6]. Though difference in average hemoglobin (Hb) A_1c_ was found to explain virtually all of the difference in risk of complications between the intensive and conventional groups in the DCCT [7], it is still under debate whether or not variability of glycemic control has also a role.

Glycemic variability comprises “glucose variability” and “HbA_1c_ variability”. Glucose variability relates to within-day fluctuations of glycemia, especially as a consequence of post-prandial hyperglycemia [8], which may eventually reflect in HbA_1c_ levels above the normal range. Conversely, HbA_1c_ variability relates to changes in glycemia over longer periods of time, which result in change in HbA_1c_ from one visit to the next [9]. Several observational studies have shown that post-prandial glucose, even in the range of impaired glucose tolerance, is a strong independent predictor of CVD [10]. However, three retrospective analyses of the DCCT found that within-day glucose variability does not contribute to the development of microvascular complications [11-13]. Moreover, a prospective study specifically addressing this issue showed no effect of either post-prandial glucose [14] or glucose variability [15] on CVD events, though a post hoc subgroup analysis demonstrated lower CVD risk in older patients targeting postprandial versus fasting/pre-meal glycemia [16] and continuous glucose monitoring showed that intraday glycemic variability is associated with the presence and severity of arterial lesions in subjects with type 2 diabetes undergoing coronary angiography for chest pain [17].

Conversely, robust evidence links HbA_1c_ variability to the risk of microvascular complications in both type 1 and type 2 diabetes. In fact, retrospective analyses of the DCCT [18] and the Finnish Diabetic Nephropathy (FinnDiane) Study [19] have shown that change in HbA_1c_ from one visit to the next is an independent risk factor for the development of both diabetic retinopathy (DR) and nephropathy (DN) in individuals with type 1 diabetes. In addition, HbA_1c_ variability was shown to be an independent variable that added to the effect of HbA_1c_ on the risk of microalbuminuria in adolescent patients with type 1 diabetes from the Oxford Regional Prospective Study and the Nephropathy Family Study [20]. Very recently, two prospective cohort studies from Japan and Taiwan, the Tsukuba Kawai Diabetes Registry 2 [21] and the Diabetes Management through an Integrated Delivery System project [22], have shown that HbA_1c_ variability is associated with microalbuminuria in patients with type 2 diabetes even after adjustment for known predictors of albuminuria. These data are consistent with our recent report from the Renal Insufficiency And Cardiovascular Events (RIACE) Italian Multicenter Study that HbA_1c_ variability is an independent correlate of albuminuria and the albuminuric phenotypes of chronic kidney disease (CKD), but not of non-albuminuric CKD and DR [23].

However, data are less stringent with respect to the association of HbA_1c_ variability with CVD. In type 1 diabetes, HbA_1c_ variability was predictive of incident CVD events in the FinnDiane Study [19], whereas HbA_1c_ variation (not variability) over time showed a stronger association with coronary artery disease than baseline values in the Pittsburgh Epidemiology of Diabetes Complications Study [24]. Recently, a prospective analysis of the Hong Kong Diabetes Registry has shown that HbA_1c_ variability is associated with incident CKD and CVD, independent of average HbA_1c_ and other confounding variables [25].

This analysis was aimed at assessing the independent association of HbA_1c_ variability with CVD, either total or by vascular bed, in the large cohort of Caucasian subjects with type 2 diabetes from the RIACE Italian Multicenter Study.

## Methods

### Study cohort

We used the data collected at the baseline visit for the RIACE Italian Multicenter Study (registered with ClinicalTrials.gov, NCT00715481; URL http://www.clinicaltrials.gov/ct2/show/NCT00715481), an observational, prospective cohort study on the impact of estimated glomerular filtration rate (eGFR) on morbidity and mortality from CVD in type 2 diabetes.

The RIACE cohort consisted of 15,933 Caucasian patients with type 2 diabetes (defined by the American Diabetes Association criteria), attending consecutively 19 hospital-based Diabetes Clinics of the National Health Service throughout Italy (see Additional file 1) in years 2007–2008. Exclusion criteria were dialysis or renal transplantation. The study protocol was reviewed by the locally appointed ethics boards. The quality and completeness of data were controlled and 160 patients were excluded due to missing or implausible values and the remaining 15,773 subjects were subsequently analyzed. Multiple HbA_1c_ values (3 to 5, mean±SD: 4.52±0.76) serially measured at intervals of 6–12 months during the 2-year period preceding the enrolment were available from 9 centers for a total 8,290 patients (52.6% of the entire cohort). CVD risk factors and complications were determined as part of the baseline assessment using standardized protocols across study centers.

### CVD risk factors

All patients underwent a structured interview in order to collect the following information: age, smoking status, known diabetes duration, current glucose-, blood pressure (BP)- and lipid-lowering treatments and anti-platelet and anti-coagulant therapy, with indication of the class of drug. Weight and height were assessed with calculation of body mass index (BMI); BP was measured with a sphygmomanometer after a 5 min of rest. Triglycerides, total and HDL cholesterol were determined by standard analytical methods; LDL cholesterol was calculated by the Friedwald formula. Hypertension was defined as systolic BP ≥140 mmHg and/or diastolic BP ≥90 mmHg and/or anti-hypertensive treatment. Dyslipidemia was defined as high (≥100 mg/dl) LDL cholesterol and/or lipid-lowering treatment.

### Complications

Prevalent CVD at baseline was assessed from medical history by recording previous documented major acute CVD events, including acute myocardial infarction (AMI), stroke, foot ulcer or gangrene, amputation, coronary, carotid, and lower limb revascularization, and surgery for aortic aneurysm. CVD events were adjudicated based on hospital discharge records or specialist visits by an *ad hoc* committee in each center [26].

The presence of CKD at baseline was assessed by albuminuria and serum creatinine. As previously reported in detail [27], albumin excretion rate (AER) was obtained from timed (24 hour) urine collections or calculated from albumin/creatinine ratio in early-morning, first-voided urine samples, in the absence of symptoms and signs of urinary tract infection or other interfering clinical conditions. Albuminuria was measured in one-to-three fresh urine samples for each patient by immunonephelometry or immunoturbidimetry and, in case of multiple measurements, the geometric mean was used for analysis. In subjects with multiple measurements (4,062 with at least two and 2,310 with three values), concordance rate between the first value and the geometric mean was >90% for all classes of albuminuria [27]. Patients were then assigned to one of the following categories of albuminuria (mg/24 hours): normoalbuminuria (AER <30), microalbuminuria (AER 30–299), or macroalbuminuria (AER ≥300). Serum (and urine) creatinine was measured by the modified Jaffe method. One to three measurements were obtained for each patients and eGFR was calculated by the four-variable Modification of Diet in Renal Disease (MDRD) Study equation, using the mean serum creatinine value in case of multiple measures, as reported in previous publications [27-29]. Patients were then assigned to one of the following categories of eGFR (mL/min/1.73 m^2^): 1 (≥90); 2 (60–89); 3 (30–59); 4 (15–29); and 5 (<15). Finally, subjects were classified as having no CKD or CKD stages 1–5, based on the presence or absence of micro or macroalbuminuria and the value of eGFR calculated by the MDRD Study equation, according to the National Kidney Foundation’s Kidney Disease Outcomes Quality Initiative [30]. Patients assigned to CKD stages (and GFR classes) 4 and 5 were pooled together. As previously reported [29], CKD patients were further classified as having one of the following CKD phenotypes: albuminuria alone (stages 1–2 CKD), reduced eGFR alone (stage ≥3 CKD without albuminuria), or both (stage ≥3 CKD with albuminuria).

The presence of DR at baseline was assessed by dilated fundoscopy. In each center, an expert ophthalmologist was asked to fill in a standardized report format and to classify DR into absent, mild, moderate or severe non-proliferative DR, proliferative DR, and maculopathy, according to the Global Diabetic Retinopathy Project Group [31]. Patients were classified based on the actual fundus appearance or the retinal disease condition which had eventually required a previous photocoagulation or surgical treatment. Based on the worst eye, patients with non-proliferative DR of mild or moderate degree were classified as having non-advanced DR, whereas those with severe non-proliferative DR or pre-proliferative DR, proliferative DR, maculopathy, or blindness were grouped into the advanced DR category [32].

### HbA_1c_ variability

HbA_1c_ was measured in each center by high performance liquid chromatography using DCCT-aligned methods. Average HbA_1c_ and HbA_1c_ variability were calculated for each patient as the intra-individual mean (HbA_1c_-MEAN) and SD (HbA_1c_-SD), respectively, for HbA_1c_ values obtained during the 2-year period preceding recruitment, including that obtained at the enrolment. The inter-individual difference in the number of HbA_1c_ assessments (a few values would make the SD apparently greater than many values) was adjusted according to the formula: adj-HbA_1c_-SD = SD/√[n/(n-1)] [9,11]. Furthermore, as a normalized measure of variability, the coefficient of variation of HbA_1c_ (HbA_1c_-CV) was calculated as the ratio of HbA_1c_-SD and HbA_1c_-MEAN to correct for larger SD due to higher absolute values of HbA_1c_-MEAN [19].

### Statistical analysis

Data are expressed as median (interquartile range, IQR) and/or mean±SD, for continuous variables, and number of cases and percentage for categorical variables. Patients were stratified by previous major acute CVD event(s). Continuous variables were compared by Student’s t test or one-way ANOVA, for normally distributed variables, and by Mann–Whitney U test or Kruskall-Wallis test, in case of variables with a skewed distribution. Pearson chi-square was applied to categorical variables.

Logistic regression analyses with backward variable selection (probability for removal >0.10) were performed to assess whether increments in HbA_1c_-MEAN (Model 1), increments of HbA_1c_-MEAN and HbA_1c_-SD (Model 2), and quartiles of both variables (Model 3) are independent correlates of CVD complications as compared with no CVD. Covariates were, age, BMI, gender, known disease duration, smoking habits, triglycerides, HDL cholesterol, hypertension, dyslipidemia, albuminuria and eGFR categories, CKD phenotype and DR stage, specific treatments and either eGFR and albuminuria categories if DR was the dependent variable, or DR categories if renal parameters were the dependent variable. Results of these analyses were expressed as odd ratios (ORs) with their 95% confidence intervals (CIs). Logistic regression analyses s were repeated entering adj-HbA_1c_-SD (or HbA_1c_-CV) instead of HbA_1c_-SD as a measure of HbA_1c_ variability.

All p values were two-sided, and a p value of less than 0.05 was considered statistically significant. Statistical analyses were performed using SPSS version 13.0 (SPSS Inc., Chicago, Illinois, USA).

## Results

### Patients’ characteristics

As previously reported [23], participants included in this analysis, i.e. those having multiple (3–5) HbA_1c_ values, had a median (IQR) age and duration of diabetes at enrolment of 68 (61–74) and 14 (7–23) years, respectively, and a male/female ratio of 57/43. Likely due to longer disease duration, these subjects showed a worse CVD risk profile and a higher prevalence of any CVD event and were more frequently on glucose-, lipid- and BP-lowering drug treatment, than those excluded from the analysis due to unavailability of serial HbA_1c_ measurements. HbA_1c_-MEAN of participants was 7.57% (6.86-8.38), HbA_1c_-SD was 0.46 (0.29-0.74) and adj-HbA_1c_-SD was 0.40 (0.25-0.65). Both variability measures, i.e. HbA_1c_-SD and adj-HbA_1c_-SD, were closely related to HbA_1c_-MEAN and between each other.

One or more major acute CVD events were adjudicated in 2,133 of the 8,290 patients analyzed (25.7%). Clinical characteristics of subjects with or without prior CVD event(s) are shown in Tables 1 and 2. As compared with individuals without CVD, patients with prior event(s) were older, predominantly male, and had a longer diabetes duration, a worse metabolic control, a significantly higher rate of insulin treatment, and a slightly but significantly higher number of HbA_1c_ measures (4.63±0.70 versus 4.48±0.78, *P*<0.0001). Concerning the other CVD risk factors, these patients were more frequently treated for dyslipidemia and/or hypertension and had higher triglycerides and lower HDL cholesterol, but also lower total, LDL and non-HDL cholesterol and (marginally) BP levels. Finally, subjects with prior CVD event(s) showed a higher prevalence of DR, albuminuria, reduced eGFR and CKD.

**Table 1:** CVD risk factors in study subjects

**Variables**	**CVD no**	**CVD yes**	***P***
**n (%)**	6,157 (74.3)	2,133 (25.7)	
**Males (%)**	3,300 (53.6)	1,426 (66.9)	<0.0001
**Age, years**	66.4±10.0	69.6±9.3	<0.0001
	67 (60–73)	70 (63–76)	
**Age at diabetes diagnosis, years**	52.0±10.6	51.2±10.9	0.003
	52 (45–59)	51 (44–58)	
**Diabetes duration, years**	14.5±10.0	18.4±10.4	<0.0001
	12 (6–21)	18 (10–26)	
**Smoking, n (%)**			<0.0001
**Never**	3,478 (56.5)	1,006 (47.2)	
**Former**	1,739 (28.2)	817 (38.3)	
**Current**	940 (15.3)	310 (14.5)	
**Current HbA**_**1c**_**, %**	7.57±1.36	7.86±1.40	<0.0001
	7.42 (6.70-8.28)	7.66 (6.92-8.59)	
**HbA**_**1c**_**-MEAN, %**	7.63±1.19	7.88±1.21	<0.0001
	7.49 (6.81-8.31)	7.78 (7.04-8.56)	
**HbA**_**1c**_**-SD, %**	0.59±0.48	0.60±0.47	0.105
	0.46 (0.28-0.73)	0.47 (0.29-0.75)	
**HbA**_**1c**_**-CV, %**	7.57±5.52	7.45±5.17	0.936
	6.06 (3.94-9.37)	6.17 (3.96-9.33)	
**Adj-HbA**_**1c**_**-SD, %**	0.52±0.42	0.53±0.41	0.058
	0.40 (0.26-0.67)	0.42 (0.26-0.67)	
**BMI, kg/m**^**2**^			
**Males**	28.2±4.3	28.6±4.5	0.017
	27.7 (25.2-30.6)	28.1 (25.4-31.0)	
**Females**	29.4±5.7	29.5±5.8	0.820
	28.7 (25.5-32.5)	28.8 (25.5-32.8)	
**Triglycerides, mmol/l**	1.52±0.90	1.58±0.94	0.016
	1.30 (0.95-1.83)	1.35 (0.98-1.92)	
**Total cholesterol, mmol/l**	4.82±0.93	4.56±0.95	<0.0001
	4.76 (4.20-5.39)	4.77 (3.91-5.12)	
**HDL cholesterol, mmol/l**			
**Males**	1.25±0.33	1.19±0.31	<0.0001
	1.22 (1.01-1.45)	1.14 (0.97-1.35)	
**Females**	1.41±0.37	1.33±0.33	<0.0001
	1.35 (1.14-1.59)	1.28 (1.09-1.52)	
**LDL cholesterol, mmol/l**	2.81±0.80	2.61±0.81	<0.0001
	2.77 (2.36-4.01)	2.54 (2.05-3.07)	
**Non-HDL cholesterol, mmol/l**	3.50±0.90	3.33±0.90	<0.0001
	3.42 (2.82-3.98)	3.22 (2.69-3.83)	
**Dyslipidemia, n (%)**	5,032 (81.7)	1,871 (87.7)	<0.0001
**Systolic BP, mmHg**	139.8±18.0	138.9±19.1	0.050
	140 (130–150)	140 (125–150)	
**Diastolic BP, mmHg**	78.9±9.1	77.4±9.4	<0.0001
	80 (71–85)	80 (70–82)	
**Hypertension, n (%)**	5,121 (83.2)	1,987 (93.2)	<0.0001

Prevalence of CVD risk factors in individuals with or without prior CVD event(s). Values are mean±SD and median (interquartile range) for continuous variables and n (%) for categorical variables; *P* values for comparison between groups using the Student’s t test for parametric or the corresponding Mann–Whitney U test for nonparametric (HbA_1c_-SD, HbA_1c_-CV, Adj- HbA_1c_-SD, and triglycerides) continuous variables, and the χ^2^ test for categorical variables.

**Table 2:** Complications and treatments in study subjects

**Variables**	**CVD no**	**CVD yes**	***P***
**n (%)**	6,157 (74.3)	2,133 (25.7)	
**Albuminuria, mg/24 hours**	60.6±275.6	125.6±554.8	<0.0001
	12.6 (6.2-28.1)	17.4 (8.1-54.0)	
**Serum creatinine, μmol/l**	82.2±31.8	94.6±39.8	<0.0001
	77.8 (65.4-90.2)	85.7 (72.5-106.1)	
**eGFR**^**MDRD**^**, ml/min/1.73 m**^**2**^	80.1±22.1	71.7±23.2	<0.0001
	79.0 (66.3-92.9)	72.2 (55.9-86.8)	
**eGFR**^**CKD-EPI**^**, ml/min/1.73 m**^**2**^	81.8±19.4	71.7±21.5	<0.0001
	84.4 (69.1-94.6)	74.8 (56.4-88.7)	
**Albuminuria**			<0.0001
**Normoalbuminuria**	4,687 (76.1)	1,366 (64.0)	
**Microalbuminuria**	1,227 (19.9)	602 (28.2)	
**Macroalbuminuria**	243 (3.9)	408 (7.7)	
**eGFR (MDRD)**			<0.0001
**≥90 ml/min/1.73 m**^**2**^	1,824 (29.6)	424 (19.9)	
**60**–**89 ml/min/1.73 m**^**2**^	3,349 (54.4)	1,051 (49.3)	
**30**–**59 ml/min/1.73 m**^**2**^	915 (14.9)	586 (27.5)	
**<30 ml/min/1.73 m**^**2**^	69 (1.1)	72(3.4)	
**CKD phenotype**			<0.0001
**no CKD**	4,081 (66.3)	1,038 (48.7)	
**stages 1–2 CKD**	1,092 (17.7)	437 (20.5)	
**stage ≥3 CKD nonalbuminuric**	606 (9.8)	328 (15.4)	
**stage ≥3 CKD albuminuric**	378 (6.1)	330 (15.5)	
**Retinopathy, n (%)**			<0.0001
**No retinopathy**	4,841 (78.6)	1,428 (66.9)	
**Non-advanced retinopathy**	841 (13.7)	429 (20.1)	
**Advanced retinopathy**	475 (7.7)	276 (12.9)	
**Any coronary event, n (%)**	—	1,373 (64.4)	—
**Acute myocardial infarction, n (%)**	—	949 (44.5)	—
**Any cerebrovascular event, n (%)**	—	902 (42.3)	—
**Stroke, n (%)**	—	257 (12.0)	—
**Any lower limb vascular event, n (%)**	—	584 (27.4)	—
**Ulceration/gangrene, n (%)**	—	319 (15.0)	—
**Diabetes treatment, n (%)**			<0.0001
**Diet**	729 (11.8)	151 (7.1)	
**OHA**	4,051 (65.8)	4,353 (59.9)	
**Insulin + OHA**	570 (9.3)	205 (9.6)	
**Insulin**	807 (13.1)	500 (23.4)	
**Lipid-lowering treatment, n (%)**	2,681 (43.5)	1,435 (67.3)	<0.0001
**Anti-hypertensive treatment, n (%)**	4,232 (68.7)	1,850 (86.7)	<0.0001
**ACE-I/ARB treatment, n (%)**	3,522 (57.2)	1,469 (68.9)	<0.0001

Prevalence of micro and macrovascular complications and type and rate of treatments in individuals with or without prior CVD event(s). Values are mean±SD and median (interquartile range) for continuous variables and n (%) for categorical variables; *P* values for comparison between groups using the Student’s t test for parametric or the corresponding Mann–Whitney U test for nonparametric (albuminuria, serum creatinine and eGFR) continuous variables, and the χ^2^ test for categorical variables.

### Average HbA_1c_ and HbA_1c_ variability

As previously reported [23], increasing HbA_1c_-MEAN and HbA_1c_-SD (or HbA_1c_-CV) were associated with longer and shorter diabetes duration, respectively, whereas both measures correlated with a more adverse CVD risk profile, more severe complications, and more intensive treatment, with a few differences.

Values for current HbA_1c_, HbA_1c_-MEAN, HbA_1c_-SD, HbA_1c_-CV and Adj-HbA_1c_-SD in subjects with or without any major acute CVD event are given in Tables 1 (total) and 3 (by vascular bed, i.e. coronary, carotid and lower limb). As compared with men, women showed significantly higher values of current HbA_1c_ (7.75±1.40 versus 7.58±1.32, *P*<0.0001), HbA_1c_-MEAN (7.80±1.24 versus 7.62±1.17, *P*<0.0001) and lower levels of HbA_1c_-SD (0.59±0.49 versus 0.60±0.47, *P*=0.049), HbA_1c_-CV (7.39±5.50 versus 7.65±5.38, *P*=0.001), and, non-significantly, Adj-HbA_1c_-SD (0.52±0.43 versus 0.52±0.40, *P*=0.060), as compared with men. When stratified by presence or absence of any CVD event, total and by vascular bed, data in men and women were almost identical to those from the whole cohort presented in Table 3 (Additional file 2: Tables S1-S2). Current HbA_1c_ and HbA_1c_-MEAN were higher in patients with history of any CVD, either total or by vascular bed, whereas HbA_1c_-SD and Adj-HbA_1c_-SD were significantly increased in subjects with AMI, any lower limb vascular event and ulceration/gangrene and HbA_1c_-CV only in those with this latter event. Current HbA_1c_ and HbA_1c_-MEAN values, but not measures of HbA_1c_ variability, increased with the number of major acute CVD events and with the number of vascular beds affected (P<0.0001). However, while these values did not change significantly with increasing number of events (from 1 to ≥5), they were higher in subjects with 2 or 3 vascular beds affected than in those with involvement of only one site.

**Table 3:** **HbA**_**1c**_**-values and measures of HbA**_**1c**_**-variability**

**Variable**	**n (%)**	**Current HbA**_**1c**_**, %**	**HbA**_**1c**_**-MEAN, %**	**HbA**_**1c**_**-SD, %**	**HbA**_**1c**_**-CV, %**	**Adj-HbA**_**1c**_**-SD, %**
**No CVD**	6,157 (74.3)	7.57±1.36	7.63±1.19	0.59±0.48	7.57±5.52	0.52±0.42
		7.42 (6.70-8.28)	7.49 (6.81-8.31)	0.46 (0.28-0.73)	6.06 (3.94-9.37)	0.40 (0.26-0.67)
**Any coronary event**	1,373 (16.6)	7.85±1.34	7.88±1.20	0.60±0.45	7.40±5.13	0.53±0.39
		7.70 (6.92-8.57)	7.77 (7.02-8.59)	0.47 (0.29-0.74)	6.18 (3.95-9.22)	0.41 (0.26-0.66)
***P***		<0.0001	<0.0001	0.194	0.821	0.140
**Acute myocardial infarction**	949 (11.4)	7.80±1.31	7.86±1.18	0.62±0.45	7.64±5.08	0.54±0.39
		7.67 (6.92-8.51)	7.78 (7.05-8.53)	0.49 (0.31-0.77)	6.39 (4.13-9.44)	0.43 (0.27-0.67)
***P***		<0.0001	<0.0001	0.004	0.068	0.003
**Any cerebrovascular event**	902 (10.9)	8,00±1.51	7.96±1.24	0.59±0.45	7.18±4.91	0.52±0.40
		7.75 (7.02-8.73)	7.83 (7.12-8.64)	0.47 (0.28-0.72)	5.91 (3.74-8.92)	0.41 (0.25-0.64)
***P***		<0.0001	<0.0001	0.956	0.103	0.672
**Stroke**	257 (3.1)	7.64±1.37	7.70±1.21	0.59±0.40	7.56±4.86	0.51±0.34
		7.45 (6.61-8.43)	7.63 (6.75-8.45)	0.48 (0.30-0.74)	6.28 (4.25-9.40)	0.42 (0.27-0.64)
***P***		0.470	0.354	0.299	0.381	0.325
**Any lower limb vascular event**	584 (7.0)	8.02±1.45	8.02±1.19	0.64±0.50	7.68±5.34	0.56±0.45
		7.85 (7.09-8.73)	7.92 (7.22-8.66)	0.50 (0.31-0.80)	6.32 (4.10-9.38)	0.45 (0.27-0.69)
***P***		<0.0001	<0.0001	0.013	0.372	0.005
**Ulceration/gangrene**	319 (3.8)	7.93±1.55	8.01±1.25	0.71±0.58	8.49±5.93	0.62±0.51
		7.67 (6.98-8.62)	7.88 (7.18-8.64)	0.54 (0.34-0.86)	6.76 (4.31-10.23)	0.48 (0.30-0.76)
***P***		<0.0001	<0.0001	<0.0001	0.004	<0.0001

Current HbA_1c_, HbA_1c_-MEAN, HbA_1c_-SD, HbA_1c_-CV, and Adj-HbA_1c_-SD values in patients with any major acute CVD event by vascular bed (coronary, carotid and lower limb) versus subjects without CVD. Values are mean±SD and median (interquartile range); *P* values for comparison between groups using the Student’s t test for parametric or the corresponding Mann–Whitney U test for nonparametric (HbA_1c_-SD, HbA_1c_-CV, and Adj- HbA_1c_-SD) variables.

### Multiple logistic regression models

We used logistic models as multivariate regression analyses because of several potential confounding factors for the association between HbA_1c_ variability and prevalence of macrovascular complications. Even after adjusting for CVD risk factors and complications, HbA_1c_-MEAN, but not HbA_1c_-SD (and independent of it in Models 2 and 3) was a significant correlate of any CVD (Table 4).

**Table 4:** Independent correlates of any CVD event(s)

**Variables**	**Any CVD event**		
	**OR**	**95%CI**	***P***
**Age, x year**	1.018	1.011-1.024	<0.0001
**Diabetes duration, x year**	1.019	1.013-1.025	<0.0001
**Gender, male**	1.746	1.550-1.967	<0.0001
**BMI, x unit**	-	-	-
**Smoking**			<0.0001
**No**	1.0		
**Former**	1.413	1.254-1.591	<0.0001
**Current**	1.156	0.986-1.354	0.074
**Triglycerides, x 0.0113 mmol/l**	0.999	0.999-1.000	0.040
**HDL cholesterol, x0.259mmol/l**	0.983	0.978-0.987	<0.0001
**Dyslipidemia**	1.713	1.470-1.997	<0.0001
**Hypertension**	2.054	1.699-2.484	<0.0001
**Diabetes treatment, n (%)**			<0.0001
**Diet**	1.0		
**OHA**	1.266	1.038-1.544	0.020
**Insulin + OHA**	1.228	0.942-1.601	0.128
**Insulin**	2.051	1.623-2.593	<0.0001
**Albuminuria**			0.052
**Normoalbuminuria**	1.0		
**Microalbuminuria**	1.167	1.030-1.323	0.015
**Macroalbuminuria**	1.069	0.845-1.351	0.580
**eGFR (MDRD)**			<0.0001
**≥90 ml/min/1.73 m**^**2**^	1.0		
**60**–**89 ml/min/1.73 m**^**2**^	1.116	0.975-1.278	0.112
**30**–**59 ml/min/1.73 m**^**2**^	1.820	1.531-2.162	<0.0001
**<30 ml/min/1.73 m**^**2**^	2.278	1.541-3.367	<0.0001
**Retinopathy**			<0.0001
**No retinopathy**	1.0		
**Non advanced retinopathy**	1.295	1.122-1.495	0.0001
**Advanced retinopathy**	1.298	1.084-1.554	0.005
**MODEL 1**			
**HbA**_**1c**_**-MEAN, 1% increment**	1.062	1.011-1.114	0.016
**MODEL 2**			
**HbA**_**1c**_**-MEAN, 1% increment**	1.062	1.011-1.114	0.016
**HbA**_**1c**_**-SD, 1% increment**	-	-	-
**MODEL 3**			
**HbA**_**1c**_**-MEAN**			0.001
**quartile 1**	1.0		
**quartile 2**	0.923	0.787-1.082	0.322
**quartile 3**	1.248	1.066-1.462	0.006
**quartile 4**	1.096	0.928-1.295	0.281
**HbA**_**1c**_**-SD quartiles**	–	–	–

Logistic regression analysis with backward variables selection of independent correlates of any CVD event versus no CVD event. ORs of variables except HbA_1c_-MEAN, HbA_1c_-SD, and their quartiles were determined by multivariate logistic analysis from MODEL 1. The results did not change significantly in MODEL 2 and MODEL 3.

When CVD was examined by vascular bed, similar findings were observed in subjects with versus those without any coronary event (AMI and/or coronary revascularization) or any cerebrovascular event (stroke and/or carotid revascularization) in all 3 models (Tables 5 and 6) and in those with versus without AMI in Model 3 only (Table 5). Conversely, none of these measures were associated with stroke (Table 6), whereas both correlated with any lower limb vascular event (ulceration/gangrene and/or lower limb revascularization; Hb-A_1c_-SD only in Model 2) and HbA_1c_-SD alone with ulceration/gangrene (Table 7).

**Table 5:** Independent correlates of coronary events

**Variables**	**Any coronary event**			**AMI**		
	**OR**	**95% CI**	***P***	**OR**	**95% CI**	***P***
**Age, x year**	1.015	1.007-1.023	0.003	1.025	1.015-1.034	<0.0001
**Diabetes duration, x year**	1.019	1.012-1.026	<0.0001	1.013	1.004-1.021	0.002
**Gender, male**	2.066	1.792-2.382	<0.0001	2.493	2.103-2.956	<0.0001
**BMI, x unit**	—	—	—	—	—	—
**Smoking**			<0.0001			<0.0001
**No**	1.0			1.0		
**Former**	1.496	1.301-1.719	<0.0001	1.560	1.327-1.834	<0.0001
**Current**	1.125	0.930-1.361	0.226	1.116	0.890-1.400	0.343
**Triglycerides, x 0.0113 mmol/l**	3.177	2.436-4.144	<0.0001	4.297	2.994-6.116	<0.0001
**HDL cholesterol, x0.259mmol/l**	0.981	0.975-0.986	<0.0001	0.982	0.976-0.988	<0.0001
**Dyslipidemia**	2.076	1.710-2.521	<0.0001	2.431	1.911-3.092	<0.0001
**Hypertension**	0.999	0.998-1.000	0.012	—	—	—
**Diabetes treatment, n (%)**			<0.0001			<0.0001
**Diet**	1.0			1.0		
**OHA**	1.205	0.953-1.524	0.119	1.302	0.988-1.716	0.061
**Insulin + OHA**	1.342	0.985-1.828	0.062	1.870	1.326-2.636	<0.0001
**Insulin**	2.013	1.528-2.652	<0.0001	2.444	1.786-3.345	<0.0001
**Albuminuria**			<0.0001	—	—	—
**Normoalbuminuria**	1.0					
**Microalbuminuria**	1.405	1.187-1.665	<0.0001			
**Macroalbuminuria**	1.146	0.839-1.565	0.390			
**eGFR (MDRD)**			<0.0001			<0.0001
**≥90 ml/min/1.73 m**^**2**^	1.0			1.0		
**60**–**89 ml/min/1.73 m**^**2**^	1.133	0.963-1.333	0.131	1.196	0.984-1.455	0.072
**30**–**59 ml/min/1.73 m**^**2**^	1.949	1.592-2.388	<0.0001	2.018	1.591-2.560	<0.0001
**<30 ml/min/1.73 m**^**2**^	1.731	1.087-2.757	0.021	1.714	1.005-2.924	0.048
**Retinopathy**			0.016			0.049
**No retinopathy**	1.0			1.0		
**Non advanced retinopathy**	1.229	1.037-1.457	0.017	1.221	1.001-1.489	0.048
**Advanced retinopathy**	1.255	1.018-1.547	0.033	1.260	0.991-1.603	0.060
**MODEL 1**						
**HbA**_**1c**_**-MEAN, 1% increment**	1.069	1.010-1.133	0.022	–	–	–
**MODEL 2**						
**HbA**_**1c**_**-MEAN, 1% increment**	1.069	1.010-1.133	0.022	–	–	–
**HbA**_**1c**_**-SD, 1% increment**	–	–	–	–	–	–
**MODEL 3**						
**HbA**_**1c**_**-MEAN**			0.004			0.003
**quartile 1**	1.0			1.0		
**quartile 2**	0.961	0.793-1.163	0.680	0.956	0.763-1.197	0.692
**quartile 3**	1.297	1.073-1.567	0.007	1.68	1.097-1.706	0.005
**quartile 4**	1.159	0.950-1.414	0.146	1.075	0.849-1.360	0.549
**HbA**_**1c**_**-SD quartiles**	–	–	–	–	–	–

Logistic regression analysis with backward variables selection of independent correlates of any coronary event and acute myocardial infarction (AMI) versus no CVD. ORs of variables except HbA_1c_-MEAN, Hb-A_1c_-SD and their quartiles were determined by multivariate logistic analysis from MODEL 1. The results did not change significantly in MODEL 2 and MODEL 3.

**Table 6:** Independent correlates of cerebrovascular events

**Variables**	**Any cerebrovascular event**			**Stroke**		
	**OR**	**95% CI**	***P***	**OR**	**95% CI**	***P***
**Age, x year**	1.022	1.012-1.032	<0.0001	1.046	1.030-1.062	<0.0001
**Diabetes duration, x year**	1.033	1.025-1.042	<0.0001	–	–	–
**Gender, male**	1.720	1.452-2.036	<0.0001	2.041	1.520-2.739	<0.0001
**BMI, x unit**	1.016	1.000-1.033	0.046	–	–	–
**Smoking**			0.060			0.020
**No**	1.0			1.0		
**Former**	1.196	1.010-1.416	0.030	1.446	1.081-1.935	0.013
**Current**	1.216	0.976-1.516	0.081	1.503	1.030-2.194	0.034
**Triglycerides, x 0.0113 mmol/l**	1.520	1.158-1.995	0.003	2.269	1.348-3.819	0.002
**HDL cholesterol, x0.259mmol/l**	0.983	0.977-0.989	<0.0001	0.987	0.977-0.998	0.017
**Dyslipidemia**	1.780	1.424-2.224	<0.0001	1.644	1.125-2.402	0.010
**Hypertension**	–	–	–	–	–	–
**Diabetes treatment, n (%)**			<0.0001			<0.0001
**Diet**	1.0			1.0		
**OHA**	1.176	0.881-1.570	0.272	1.339	0.808-2.218	0.257
**Insulin + OHA**	0.611	0.402-0.927	0.021	1.756	0.938-3.297	0.080
**Insulin**	1.911	1.375-2.657	<0.0001	3.541	2.074-6.046	<0.0001
**Albuminuria**			<0.0001	–	–	–
**Normoalbuminuria**	1.0					
**Microalbuminuria**	1.405	1.187-1.665	<0.0001			
**Macroalbuminuria**	1.146	0.839-1.565	0.390			
**eGFR (MDRD)**			<0.0001			0.001
**≥90 ml/min/1.73 m**^**2**^	1.0			1.0		
**60**–**89 ml/min/1.73 m**^**2**^	1.126	0.211.376	0.247	1.394	0.963-2.018	0.078
**30**–**59 ml/min/1.73 m**^**2**^	1.747	1.367-2.233	<0.0001	1.817	1.167-2.830	0.008
**<30 ml/min/1.73 m**^**2**^	2.759	1.718-4.431	<0.0001	3.798	1.868-7.721	<0.0001
**Retinopathy**			<0.0001	–	–	–
**No retinopathy**	1.0					
**Non advanced retinopathy**	1.458	1.207-1.762	<0.0001			
**Advanced retinopathy**	1.089	0.840-1.411	0.521			
**MODEL 1**						
**HbA**_**1c**_**-MEAN, 1% increment**	1.096	1.026-1.171	0.007	–	–	–
**MODEL 2**						
**HbA**_**1c**_**-MEAN, 1% increment**	1.096	1.026-1.171	0.007	—	—	—
**HbA**_**1c**_**-SD, 1% increment**	—	—	—	—	—	—
**MODEL 3**						
**HbA**_**1c**_**-MEAN**			0.002	—	—	—
**quartile 1**	1.0					
**quartile 2**	00.981	0.776-1.241	0.874			
**quartile 3**	1.410	1.116-1.781	0.004			
**quartile 4**	1.325	1.029-1.707	0.029			
**HbA**_**1c**_**-SD quartiles**	–	–	–	–	–	–

Logistic regression analysis with backward variables selection of independent correlates of any cerebrovascular event and stroke versus no CVD. ORs of variables except HbA_1c_-MEAN, Hb-A_1c_-SD and their quartiles were determined by multivariate logistic analysis from MODEL 1. The results did not change significantly in MODEL 2 and MODEL 3.

**Table 7:** Independent correlates of lower limb vascular events

**Variables**	**Any lower limb vascular event**			**Ulceration/gangrene**		
	**OR**	**95% CI**	***P***	**OR**	**95% CI**	***P***
**Age, x year**	1.025	1.013-1.037	<0.0001	1.033	1.017-1.048	<0.0001
**Diabetes duration, x year**	1.033	1.023-1.043	<0.0001	1.024	1.011-1.037	<0.0001
**Gender, male**	1.727	1.408-2.118	<0.0001	1.448	1.123-1.866	0.004
**BMI, x unit**	–	–	–	–	–	–
**Smoking**			0.008	–	–	–
**No**	1.0					
**Former**	1.347	1.100-1.650	0.004			
**Current**	1.317	1.010-1.718	0.042			
**Triglycerides, x 0.0113 mmol/l**	1.385	1.005-1.909	0.047	–	–	–
**HDL cholesterol, x0.259mmol/l**	0.979	0.972-0.987	<0.0001	0.985	0.976-0.994	0.001
**Dyslipidemia**	1.557	1.201-2.018	0.001	–	–	–
**Hypertension**	–	–	–	–	–	–
**Diabetes treatment, n (%)**			<0.0001			<0.0001
**Diet**	1.0			1.0		
**OHA**	1.274	0.871-1.861	0.212	1.828	1.023-3.266	0.042
**Insulin + OHA**	1.146	0.710-1.848	0.577	1.669	0.845-3.296	0.140
**Insulin**	2.014	1.321-3.072	0.001	2.948	1.595-5.449	0.001
**Albuminuria**			<0.0001			<0.0001
**Normoalbuminuria**	1.0			1.0		
**Microalbuminuria**	1.527	1.248-1.868	<0.0001	1.878	1.447-2.436	<0.0001
**Macroalbuminuria**	1.375	0.967-1.957	0.076	2.053	1.351-3.119	0.001
**eGFR (MDRD)**			<0.0001			<0.0001
**≥90 ml/min/1.73 m**^**2**^	1.0			1.0		
**60**–**89 ml/min/1.73 m**^**2**^	1.221	0.947-1.574	0.124	1.195	0.851-1.678	0.303
**30**–**59 ml/min/1.73 m**^**2**^	2.187	1.626-2.942	<0.0001	2.104	1.430-3.097	<0.0001
**<30 ml/min/1.73 m**^**2**^	2.001	1.107-3.618	0.022	2.059	1.018-4.165	0.044
**Retinopathy**			<0.0001			<0.0001
**No retinopathy**	1.0			1.0		
**Non advanced retinopathy**	1.696	1.353-2.125	<0.0001	1.880	1.403-2.520	<0.0001
**Advanced retinopathy**	1.892	1.439-2.489	<0.0001	2.680	1.926-3.729	<0.0001
**MODEL 1**						
**HbA**_**1c**_**-MEAN, 1% increment**	1.086	1.003-1.175	0.042	–	–	–
**MODEL 2**						
**HbA**_**1c**_**-MEAN, 1% increment**	1.086	1.003-1.175	0.042	–	–	
**HbA**_**1c**_**-SD, 1% increment**	1.208	0.974-1.497	0.085	1.407	1.134-1.746	0.036
**MODEL 3**						
**HbA**_**1c**_**-MEAN**			0.026	–	–	–
**quartile 1**	1.0					
**quartile 2**	1.075	0.800-1.445	0.632			
**quartile 3**	1.466	1.101-1.953	0.009			
**quartile 4**	1.296	0.967-1.737	0.083			
**HbA**_**1c**_**-SD quartiles**	–	–	–			0.036
**quartile 1**				1.0		
**quartile 2**				1.057	0.736-1.518	0.764
**quartile 3**		–		1.018	0.709-1.462	0.922
**quartile 4**				1.498	1.061-2.116	0.022

Logistic regression analysis with backward variables selection of independent correlates of any lower limb vascular event and ulceration/gangrene versus no CVD. ORs of variables except HbA_1c_-MEAN, Hb-A_1c_-SD and their quartiles were determined by multivariate logistic analysis from MODEL 1. The results did not change significantly in MODEL 2 and MODEL 3.

In all models, expressing HbA_1c_ variability as HbA_1c_-CV or adj-HbA_1c_-SD instead of HbA_1c_-SD did not change the results.

## Discussion

Recently, microvascular complications were shown to be predicted by HbA_1c_ variability from one visit to the next, independently of average HbA_1c_ and known risk factors for microangiopathy, both in type 1 [18-20] and type 2 [21-23] diabetes. Less clear is the relation of HbA_1c_ variability with CVD. This cross-sectional analysis did not show a significant association of CVD with HbA_1c_ variability, whereas events were independently associated with average HbA_1c_. However, we did also analyze acute major CVD events occurring in different vascular beds and found significant differences in their relation with HbA_1c_ variability versus average HbA_1c_.

The correlation of average HbA_1c_ with CVD is consistent with data from large trials [5,6]; this correlation was observed in both sexes, though women had higher average HbA_1c_ values than men, in keeping with previous reports [33,34]. Conversely, the lack of association of HbA_1c_ variability with total CVD events (as well as with any coronary or cerebrovascular events, AMI or stroke) is at variance with results of previous longitudinal studies in Finnish individuals with type 1 diabetes [19] and subjects with type 2 diabetes from the Hong Kong Diabetes Registry [25]. Though this discrepancy might be explained by differences in type of diabetes or ethnicity, it is more likely attributable to the much lower HbA_1c_ variability observed in our cohort, as compared with these previous reports. In fact, HbA_1c_-SD values were 0.47 and 0.46 in subjects without and with CVD, respectively, versus 0.79 and 0.87 in the type 1 Finnish cohort [19] and 1.1 and 1.4 in the type 2 Chinese cohort [25]. Conversely, HbA_1c_-MEAN values in our cohort (7.5 and 7.8, respectively) were similar to those found in subjects from the Hong Kong Diabetes Registry (7.4 and 7.8, respectively) [25]. Thus, it might be hypothesized that the contribution of small changes in HbA_1c_ values from one visit to the next is negligible and that the impact of average HbA_1c_ would be predominant under these conditions, with values above 7.5% conferring an increased risk. In this view, the results of our study may not be in contrast with those of the above-mentioned reports [19,25]; however, if we consider that such a small variability of HbA_1c_ was significantly associated with CKD (especially albuminuric), but not DR [23], we might conclude that DN is the most sensitive complication to changes in HbA_1c_, whereas CVD and DR are influenced by wider fluctuations of this parameter over time.

The diverging correlation of acute major CVD events occurring in different vascular beds with HbA_1c_ variability versus average HbA_1c_ might reflects distinct characteristics of vascular disease at these levels. In particular, multiple mechanisms contribute to various extent to the development of ulceration/gangrene, including macroangiopathy, but also microvascular disease and neuropathy. This might explain the fact that both average Hb-A_1c_ and Hb-A_1c_ variability were associated with lower limb vascular event(s) (including also lower limb revascularization) and particularly the correlation of Hb-A_1c_ variability, but not average Hb-A_1c_, with ulceration/gangrene alone. This finding is also consistent with our previous report that the independent association of reduced eGFR with coronary events is stronger than that with peripheral events, which are related predominantly with the albuminuric CKD phenotypes reflecting predominantly microvascular disease [26].

Strengths of this study include the large size of the cohort, the completeness of data, the analysis of a contemporary dataset, the adjustment for treatments, and, as mentioned above, the analysis by vascular bed. The main limitation is the cross-sectional design for the assessment of prevalent CVD, which did not allow to examine the effect of HbA_1c_.variability on the development of macrovascular complications in uncomplicated individuals, as in the studies of Waden et al. [19] and Luk et al. [25]. Another limitation might be that the RIACE participants who had serial (3–5) HbA_1c_ measures differed significantly (i.e. longer diabetes duration, worse CVD risk profile, higher prevalence of any CVD event, and higher rate of treatment) from those who did not and were therefore excluded from this analysis. Thus, though virtually all subjects from the 9 centers which made available these data had more than two HbA_1c_ measures, independently of their HbA_1c_ variability, we cannot rule out a selection bias. Other possible limitations concerning HbA_1c_ values are that they were performed in each center as a part of the patient’s standard care with no pre-specified intervals between HbA_1c_ measurements, and that the number of measures per individual varied from 3 to 5. However, non-centralized measurements did not affect intra-individual variability, intervals between measurements ranged from 6 to 9 months, and adj-HbA_1c_-SD was used to account for difference in the number of measures. Furthermore, though the number of measurements was not as large as in the study of Sugawara et al. [21] and the period analyzed was not as long as in the study of Hsu et al. [22], re-analyses of these surveys showed that using 3-monthly (i.e. 4 to 5) HbA_1c_ measurements [21] or a series of 2-year HbA_1c_ values [22], as in our study, did not change the results. Finally, potential limitations concerning non-centralized assessment of DN and DR have been addressed in previous RIACE reports [27-29,32].

In conclusion, in patients with type 2 diabetes, HbA_1c_ variability has not a great impact on macrovascular complications, at variance with average HbA_1c_, an opposite finding as compared with microvascular disease, and particularly DN and especially albuminuric CKD phenotypes.

## Abbreviations

DCCT: Diabetes Control and Complications Trial; CVD: Cardiovascular disease; HbA1c: Hemoglobin A_1c_; FinnDiane: Finnish Diabetic Nephropathy; DR: Diabetic retinopathy; DN: Diabetic nephropathy; RIACE: Renal Insufficiency And Cardiovascular Events; CKD: Chronic kidney disease; eGFR: Estimated glomerular filtration rate; BP: Blood pressure; BMI: Body mass INDEX; AMI: Acute myocardial infarction; AER: Albumin excretion rate; MDRD: Modification of Diet in Renal Disease; HbA1c-MEAN: Average HbA_1c_; HbA1c-SD: HbA_1c_ variability; adj-HbA1c-SD: Adjusted HbA_1c_-SD; HbA1c-CV: Coefficient of variation of HbA_1c_; IQR: Interquartile range; ORs: Odd ratios; CIs: Confidence intervals.

## Competing interests

The authors declare that they have no competing interests.

## Authors’ contributions

All Authors have made substantial contributions to conception and design, or acquisition of data, or analysis and interpretation of data; have been involved in drafting the manuscript or revising it critically for important intellectual content; and have given final approval of the version to be published. All authors read and approved the final manuscript.

## Supplementary Material

Additional file 1 A complete list of the RIACE Investigators can be found as Additional files 2. Click here for file

Additional file 2: Table S1 HbA_1c_-values and measures of HbA_1c_-variability in female subjects (n. 3,564). Current HbA_1c_, HbA_1c_-MEAN, HbA_1c_-SD, HbA_1c_-CV, and Adj-HbA_1c_-SD values in patients with any major acute CVD event by vascular bed (coronary, carotid and lower limb) versus subjects without CVD. **Table S2:** HbA_1c_-values and measures of HbA_1c_-variability in male subjects (n. 4,726). Current HbA_1c_, HbA_1c_-MEAN, HbA_1c_-SD, HbA_1c_-CV, and Adj-HbA_1c_-SD values in patients with any major acute CVD event by vascular bed (coronary, carotid and lower limb) versus subjects without CVD. Click here for file
