# Supplementary material for: Hemoglobin A1c variability as an independent correlate of cardiovascular disease in patients with type 2 diabetes: a cross-sectional analysis of the Renal Insufficiency and Cardiovascular Events (RIACE) Italian Multicenter Study
Source: Cardiovasc Diabetol. 2013 Jul 5;12:98. doi: 10.1186/1475-2840-12-98 (PMC3750360; doi:10.1186/1475-2840-12-98)
Supplement: Additional file 1 — A complete list of the RIACE Investigators can be found as Additional files 2. [file 1475-2840-12-98-S1.doc]

**The RIACE Study Group**

**The RIACE Steering Committee**

Giuseppe Pugliese (Coordinator), Giuseppe Penno (Secretariat), Anna Solini, Enzo Bonora, Emanuela Orsi, Roberto Trevisan, Luigi Laviola, Antonio Nicolucci.

**The Diabetic Nephropathy Study Group, SID**

Giuseppe Pugliese, Salvatore De Cosmo, Gabriella Gruden, Susanna Morano, Giuseppe Penno, Francesco Pugliese, Giampaolo Zerbini, Luigi Laviola, Anna Solini, Roberto

Trevisan.

**Participating diabetes centres**

1. Azienda Ospedaliera Sant'Andrea, Roma (Coordinating Center): Giuseppe Pugliese, Laura Salvi, Lucilla Bollanti and Alessandra Bazuro.
2. Ospedale Le Molinette, Torino: Paolo Cavallo-Perin, Gabriella Gruden and Bartolomeo Lorenzati.
3. Ospedale San Luigi Gonzaga, Orbassano: Mariella Trovati, Giovanni Anfossi†, Franco Cavalot and Massimo Chirio.
4. Ospedale San Raffaele, Milan: Giampaolo Zerbini and Valentina Martina.
5. IRCCS “Cà Granda – Ospedale Maggiore Policlinico”, Milan: Emanuela Orsi, Laura Montefusco and Dario Zimbalatti.
6. Ospedale San Paolo, Milan: Antonio Pontiroli, Annamaria Veronelli and Barbara Zecchini.
7. Ospedale San Giuseppe, Milan: Maura Arosio and Alessia Dolci.
8. Ospedali Riuniti, Bergamo: Roberto Trevisan and Anna Corsi.
9. Ospedale Maggiore, Verona: Enzo Bonora and Giacomo Zoppini.
10. Policlinico Universitario, Padova: Angelo Avogaro, Monica Vedovato and Elisa Pagnin.
11. Ospedale Cisanello, Azienda Ospedaliero-Universitaria Pisana, Pisa: Giuseppe Penno, Laura Pucci, Daniela Lucchesi, Eleonora Russo and Monia Garofolo.
12. Ospedale Santa Chiara, Azienda Ospedaliero-Universitaria Pisana, Pisa: Anna Solini.
13. Ospedale Le Scotte, Siena: Francesco Dotta, Cecilia Fondelli and Laura Nigi.
14. Policlinico Umberto I, Roma: Susanna Morano and Alessandra Gatti.
15. Ospedale S. Maria Goretti, Latina: Raffaella Buzzetti.
16. Ospedali Riuniti, Foggia: Mauro Cignarelli, Olga Lamacchia and Sabina Pinnelli.
17. Policlinico Universitario, Bari: Francesco Giorgino, Luigi Laviola and Sebastio Perrini.
18. Policlinico Mater Domini, Catanzaro: Giorgio Sesti and Francesco Andreozzi.
19. Policlinico Monserrato, Cagliari: Marco Giorgio Baroni and Giuseppina Frau.

† Dr Anfossi is deceased.
