# Supplementary material for: Hemoglobin A1c variability as an independent correlate of cardiovascular disease in patients with type 2 diabetes: a cross-sectional analysis of the Renal Insufficiency and Cardiovascular Events (RIACE) Italian Multicenter Study
Source: Cardiovasc Diabetol. 2013 Jul 5;12:98. doi: 10.1186/1475-2840-12-98 (PMC3750360; doi:10.1186/1475-2840-12-98)
Supplement: Additional file 2: Table S1 — HbA1c-values and measures of HbA1c-variability in female subjects (n. 3,564). Current HbA1c, HbA1c-MEAN, HbA1c-SD, HbA1c-CV, and Adj-HbA1c-SD values in patients with any major acute CVD event by vascular bed (coronary, carotid and lower limb) versus subjects without CVD. Table S2: HbA1c-values and measures of HbA1c-variability in male subjects (n. 4,726). Current HbA1c, HbA1c-MEAN, HbA1c-SD, HbA1c-CV, and Adj-HbA1c-SD values in patients with any major acute CVD event by vascular bed (coronary, carotid and lower limb) versus subjects without CVD. [file 1475-2840-12-98-S2.doc]

**Supplemental Table 1. HbA_1c_-values and measures of HbA_1c_-variability in female subjects (n. 3,564).** Current HbA_1c_, HbA_1c_-MEAN, HbA_1c_-SD, HbA_1c_-CV, and Adj-HbA_1c_-SD values in patients with any major acute CVD event by vascular bed (coronary, carotid and lower limb) versus subjects without CVD.

| **Variable** | **n (%)** | **Current HbA_1c_, %** | **HbA_1c_-MEAN, %** | **HbA_1c_-SD, %** | **HbA_1c_-CV, %** | **Adj-HbA_1c_-SD, %** |
| --- | --- | --- | --- | --- | --- | --- |
| **No CVD** | 2,857 (80.2) | 7.67±1.37  7.48 (6.76-8.38) | 7.73±1.22  7.59 (6.87-8.39) | 0.59±0.49  0.44 (0.28-0.73) | 7.41±5.51  5.89 (3.89-9.16) | 0.52±0.42  0.39 (0.25-0.63) |
| **Any coronary event** | 409 (11.5) | 8.09±1.45  7.85 (7.12-8.84) | 8.10±1.28  7.90 (7.22-8.81) | 0.59±0.48  0.46 (0.28-0.74) | 7.14±5.40  5.89 (3.69-8.79) | 0.52±0.42  0.41 (0.25-0.66) |
| ***p*** |  | <0.0001 | <0.0001 | 0.631 | 0.394 | 0.519 |
| **Acute myocardial infarction** | 251 (7.0) | 7.96±1.40  7.85 (7.02-8.75) | 8.01±1.24  7.90 (7.18-8.77) | 0.63±0.49  0.51 (0.31-0.77) | 7.64±5.48  6.39 (3.97-9.32) | 0.55±0.42  0.44 (0.28-0.67) |
| ***p*** |  | 0.001 | <0.0001 | 0.055 | 0.227 | 0.042 |
| **Any cerebrovascular event** | 311 (8.7) | 8.23±1.60  8.07 (7.20-9.05) | 8.17±1.29  8.01 (7.22-8.93) | 0.60±0.47  0.48 (0.28-0.74) | 7.10±4.87  6.10 (3.65-8.64) | 0.53±0.42  0.43 (0.25-0.66) |
| ***p*** |  | <0.0001 | <0.0001 | 0.565 | 0.449 | 0.390 |
| **Stroke** | 78 (2.2) | 7.81±1.36  7.80 (6.84-8.73) | 7.90±1.28  7.77 (6.80-8.81) | 0.56±0.37  0.45 (0.28-0.69) | 7.09±4.70  6.14 (3.67-8.42) | 0.49±0.32  0.41 (0.25-0.60) |
| ***p*** |  | 0.354 | 0.222 | 0.944 | 0.770 | 0.884 |
| **Any lower limb vascular event** | 193 (5.4) | 7.96±1.60  7.64 (6.99-8.81) | 8.04±1.29  7.92 (7.17-8.83) | 0.65±0.60  0.49 (0.28-0.75) | 7.72±6.02  6.26 (3.90-8.99) | 0.57±0.53  0.44 (0.25-0.67) |
| ***p*** |  | 0.004 | 0.001 | 0.318 | 0.784 | 0.232 |
| **Ulceration/gangrene** | 120 (3.4) | 7.84±1.70  7.55 (6.87-8.75) | 7.99±1.36  7.84 (6.96-8.80) | 0.72±0.69  0.50 (0.31-0.83) | 8.49±6.60  6.46 (4.03-10.12) | 0.63±0.61  0.45 (0.27-0.72) |
| ***p*** |  | 0.004 | 0.001 | 0.097 | 0.178 | 0.077 |

Values are mean±SD and median (interquartile range); * *P* values for comparison between groups using the Student’s t test for parametric or the corresponding Mann-Whitney U test for nonparametric (HbA_1c_-SD, HbA_1c_-CV, and Adj- HbA_1c_-SD) variables.

**Supplemental Table 2. HbA_1c_-values and measures of HbA_1c_-variability in male subjects (n. 4,726).** Current HbA_1c_, HbA_1c_-MEAN, HbA_1c_-SD, HbA_1c_-CV, and Adj-HbA_1c_-SD values in patients with any major acute CVD event by vascular bed (coronary, carotid and lower limb) versus subjects without CVD.

| **Variable** | **n (%)** | **Current HbA_1c_, %** | **HbA_1c_-MEAN, %** | **HbA_1c_-SD, %** | **HbA_1c_-CV, %** | **Adj-HbA_1c_-SD, %** |
| --- | --- | --- | --- | --- | --- | --- |
| **No CVD** | 3,300 (69.8) | 7.49±1.30  7.32 (6.60-8.18) | 7.55±1.16  7.42 (7.75-8.22) | 0.60±0.48  0.47 (0.29-0.74) | 7.71±5.53  6.18 (4.01-9.58) | 0.52±0.41  0.41 (0.25-0.65) |
| **Any coronary event** | 964 (20.4) | 7.75±1.28  7.64 (6.87-8.43) | 7.79±1.15  7.70 (6.96-8.48) | 0.60±0.44  0.47 (0.30-0.75) | 7.51±5.01  6.24 (4.04-9.40) | 0.53±0.38  0.42 (0.26-0.66) |
| ***p*** |  | <0.0001 | <0.0001 | 0.359 | 0.812 | 0.305 |
| **Acute myocardial infarction** | 698 (14.8) | 7.75±1.28  7.64 (6.89-8.40) | 7.81±1.15  7.73 (7.01-8.46) | 0.61±0.44  0.49 (0.31-0.77) | 7.65±4.94  6.37 (4.16-9.51) | 0.54±0.38  0.43 (0.27-0.68) |
| ***p*** |  | <0.0001 | <0.0001 | 0.063 | 0.376 | 0.054 |
| **Any cerebrovascular event** | 591 (12.5) | 7.87±1.44  7.64 (6.93-8.51) | 7.85±1.20  7.77 (7.02-8.46) | 0.58±0.45  0.45 (0.28-0.72) | 7.22±4.94  5.88 (3.91-8.97) | 0.52±0.40  0.40 (0.25-0.64) |
| ***p*** |  | <0.0001 | <0.0001 | 0.599 | 0.074 | 0.786 |
| **Stroke** | 179 (3.8) | 7.56±1.38  7.42 (6.55-8.30) | 7.62±1.17  7.61 (6.70-8.28) | 0.60±0.41  0.49 (0.32-0.76) | 7.77±4.92  6.66 (4.58-9.60) | 0.52±0.36  0.43 (0.28-0.67) |
| ***p*** |  | 0.517 | 0.450 | 0.287 | 0.317 | 0.340 |
| **Any lower limb vascular event** | 391 (8.3) | 8.04±1.37  7.93 (7.11-8.64) | 8.01±1.15  7.94 (7.24 (8.62) | 0.63±0.45  0.52 (0.31-0.81) | 7.66±4.97  6.36 (4.22-9.60) | 0.56±0.40  0.46 (0.27-0.71) |
| ***p*** |  | <0.0001 | <0.0001 | 0.031 | 0.553 | 0.016 |
| **Ulceration/gangrene** | 199 (4.2) | 7.98±1.46  7.85 (7.09-8.59) | 8.01±1.18  7.90 (7.24-8.61) | 0.70±0.50  0.56 (0.35-0.87) | 8.50±5.51  6.96 (4.57-10.24 | 0.62±0.45  0.49 (0.31-0.77) |
| ***p*** |  | <0.0001 | <0.0001 | 0.001 | 0.014 | <0.0001 |

Values are mean±SD and median (interquartile range); * *P* values for comparison between groups using the Student’s t test for parametric or the corresponding Mann-Whitney U test for nonparametric (HbA_1c_-SD, HbA_1c_-CV, and Adj- HbA_1c_-SD) variables.
